# The Involvement of *β*-Catenin/COX-2/VEGF Axis in NMDA-Caused Retinopathy

**DOI:** 10.1155/2017/9760501

**Published:** 2017-10-12

**Authors:** Dan Ning, Wei Kevin Zhang, Han Tian, Xiao-Jun Li, Min Liu, Yu-Sang Li, He-Bin Tang

**Affiliations:** ^1^Department of Pharmacology, School of Pharmaceutical Sciences, South-Central University for Nationalities, No.182, Minyuan Road, Wuhan 430074, China; ^2^Chongqing Center for Drug Evaluation and Certification, No. 76 Changjiang Yi Lu, Yuzhong District, Chongqing 400042, China; ^3^Research Institute of Huazhong University of Science and Technology in Shenzhen, Shenzhen 518057, China

## Abstract

NMDA, a molecule that is capable of producing the loss of retinal ganglia cells (RGCs), has been widely studied; however, the detailed mechanism is not yet clarified. Previously, Wnt/*β*-catenin signaling has been suggested to be involved in the NMDA-induced retinopathy. In addition, previous investigations in our group demonstrated the presence of a Wnt/*β*-catenin/COX-2 axis in dorsal root ganglions (DRGs). Therefore, here in this paper, we tested whether there is an association of such axis with NMDA-induced RGC loss. Rat retinal damage models generated by intravitreal injection of NMDA were used to measure the expression levels of *β*-catenin, COX-2, and VEGF in retinas, and the neuron numbers of the retinal GCL of rats were counted. Then, pharmacological tools (MK801, a NMDA receptor inhibitor; Dickkopf homolog 1, a specific inhibitor of the Wnt pathway; NS-398, a COX-2 inhibitor; and bevacizumab, IVB, a VEGF inhibitor) were introduced to evaluate the detailed roles of Wnt/*β*-catenin, COX-2, and VEGF in retinopathy of rats. Results demonstrated that all three factors in sequence are positively regulated neuronal loss induced by NMDA. These observations indicated that the Wnt pathway/COX-2/VEGF axis plays a pathogenic role in retinopathy and represented novel therapeutic targets.

## 1. Introduction

Retinal ganglion cell death is a characteristic of many ophthalmological diseases, such as glaucoma, proliferative diabetic retinopathy, and retinal vein occlusion [1], but the underlying mechanism is not completely clarified, while glutamate-induced neurotoxicity is confirmed [2, 3]. The N-methyl-d-aspartic acid (NMDA) receptor, a glutamate receptor subtype [4, 5], being activated can lead to a large Ca^2+^ influx. This excess intracellular Ca^2+^ causes predominant neuronal excitotoxicity mechanisms and is thought to be an underlying mechanism of glaucoma-induced neuronal cell death [6].

Recent evidence indicates that the canonical Wnt pathway was activated with the NMDA receptor activation [7, 8]. *β*-Catenin is an essential downstream effector in the canonical Wnt/*β*-catenin signaling pathway. In the absence of Wnt ligands, *β*-catenin is phosphorylated by a protein complex containing glycogen synthase kinase-3*β* (GSK-3*β*) and is constantly degraded to prevent its accumulation [9]. Upon exposure to an appropriate stimulus, *β*-catenin is translocated from the cytoplasm to the nucleus, where it interacts with members of the T-cell factor for DNA binding and regulates the expression of target genes, including inflammatory factors, such as COX-2 [10]. Retinal inflammation is believed to play a causative role in vascular leakage, which can lead to diabetic macular edema, and in retinal neovascularization. Leukostasis is believed to contribute to capillary nonperfusion and local ischemia, which subsequently induces the overexpression of vascular endothelial growth factor (VEGF) [11–14]. Increased VEGF levels are responsible for the retinal vascular leakage.

In the present study, we used NMDA-treated rats to examine the possible role of *β*-catenin accumulation on the loss of neurons in the damaged retinas of model animals. The Wnt/*β*-catenin pathway in the rat retina was further mimicked by treatment with TWS119 (a GSK-3*β* inhibitor), which caused abnormal *β*-catenin accumulation [15]. We subsequently investigated whether MK801 (a NMDA receptor inhibitor), Dickkopf-1 (DKK-1, an inhibitor of the Wnt/*β*-catenin pathway), NS-398 (a COX-2 inhibitor), and bevacizumab (IVB, a VEGF inhibitor) can modulate *β*-catenin, COX-2, and VEGF expression levels and the neuronal loss in the retinas of the rats.

## 2. Materials and Methods

### 2.1. Animals

Experimental male Sprague-Dawley rats (weighing 180–200 g) were provided by Hubei Center for Disease Control and Prevention (China). The animals were acclimatized to the laboratories for one week prior to manipulation and were group housed (except for during the experimental procedures) in a controlled environment (temperature 20 ± 2°C and humidity 70%) under a 12-hour light-dark cycle. Water and food were supplied ad libitum. All experiments followed the WHO Guidance of Humane Care and Use of Laboratory Animals. The protocols were approved by the Committee on the Ethics of Animal Experiments of the South-Central University for Nationalities, China (permit number: 2013-SCUEC-AEC-006). Every effort was made to minimize the number of animals used and their suffering.

### 2.2. Treatments of Rats

The rats were anesthetized with an intraperitoneal injection of sodium pentobarbital (Sigma Chemical, St Louis, MO, USA; 40 mg/kg). NMDA (Sigma Chemical; 2 *μ*l, 40 nM) was intravitreally injected with a 33 gauge needle connected to a microsyringe after dilation of the rat pupil with tropicamide (Sigma Chemical) in both eyes of each animal. MK801 (Sigma Chemical, St Louis, MO, USA; 0.01, 0.1, 1 *μ*M), a NMDA receptor antagonist; NS-398 (1, 10, and 100 *μ*Μ), a COX-2 selective inhibitor; DKK-1 (R&D Systems; 10, 50, 100 ng/ml), a Wnt/*β*-catenin signaling inhibitor; and bevacizumab (0.25, 2.5, and 25 mg/ml), an anti-VEGF antibody were intravitreally injected with the 80 nmol NMDA in some rats. Besides, TWS119 (Cayman Chemical, Ann Arbor, MI; 2 *μ*l, 1 *μ*Μ) was intravitreally injected after dilation of the rat pupil with tropicamide. IVB (0.25–25 mg/ml) was intravitreally injected with 1 *μ*Μ TWS119 at the same rate. NS398 (a COX-2 selective inhibitor; 1–100 *μ*Μ) was intravitreally injected with 1 *μ*Μ TWS119 in the other rats [16]. Control rats (*n* = 4) were injected with phosphate-buffered saline (PBS). The tip of the needle was inserted through the dorsal limbus of the eye [17], and the total injection volume was 2 *μ*l. Animals with lens damage or vitreal haemorrhage were excluded from the study. The rats were sacrificed on the seventh day after treatments. The eyes were enucleated, formalin-fixed for 24 h, and paraffin-embedded for sectioning.

### 2.3. Slide Sectioning and HE Staining to Count the Number of Neurons

The slides made from the eyes of the rats were stained with hematoxylin and eosin (H&E). The number of neurons in the RGC layer within an area (20 *μ*m, parallel to the retinal GCL) of the central retina (1-2 mm from the optic disc) was counted in individual sections. A total of six sections from every five serial sections were examined for each retina. The morphological characteristics of adjacent tissue sections were assessed using a Nikon Eclipse Ti fluorescence microscope (Osaka, Japan).

### 2.4. Immunohistochemistry Analysis for the Expressions of COX-2 and VEGF in Eyes

Sections of the eyes from the rats were prepared for immunohistochemical staining according to the methods described previously (with slight modifications). Samples were treated according to a simple immunohistochemical staining method using a Histofine rat MAX-PO (MULTI) kit (Nichirei Corp, Tokyo, Japan) [18]. Briefly, the sections were immersed in the dimethylbenzene for 30 min to remove paraffin, washed step elution by aqueous ethanol for 5 min each time. Then, the endogenous peroxidases were inhibited with 0.3% hydrogen peroxidase for 15 min and washed with ultrapure water. After that, the sections were treated by microwaving them in 0.01 M citrate acid buffer (pH 6.0) at 700 W for 10 min. After washing thoroughly, the sections were incubated with an anti-VEGF polyclonal antibody (Boster, Wuhan, China; 1 : 100 dilution) and anti-COX-2 polyclonal antibody (Cayman Chemical, Ann Arbor, MI; 1 : 200 dilution) overnight at 4°C. Then, the sections were immersed in DAB (Histofine, Nichirei Corp, Tokyo, Japan) and quantified by a multispectral image analysis.

### 2.5. Immunofluorescence Analysis for the Expressions of *β*-Catenin in Eyes from Rats

The immunofluorescence analysis of *β*-catenin expression was performed with an anti-*β*-catenin polyclonal antibody (Cayman; 1 : 100 dilution). The tissue sections were then incubated for 1 h at room temperature with Alexa Fluor 546 goat anti-rabbit IgG (Molecular Probes, Eugene, OR, USA; 1 : 1000 dilution), washed three times with PBS, and visualized under a Nikon Eclipse Ti fluorescence microscope [18].

### 2.6. Statistical Analysis

In the immunohistochemical analysis, the retinal layers within an area (20 *μ*m, parallel to the retinal GCL) of the central retina (1-2 mm from the optic disc) in individual sections were observed. A total of six sections from every five serial sections were examined for each retina. The data are presented as the means ± SEM. All statistical analyses were performed using GraphPad Prism 5.0 software package. Comparisons between the mean variables of two groups were made by one-way ANOVA of Bonferroni. ^†^*P* > 0.05, ^∗^*P* < 0.05, ^∗∗^*P* < 0.01, and ^∗∗∗^*P* < 0.001 versus control ^‡^*P* > 0.05, ^#^*P* < 0.05, ^##^*P* < 0.01, and ^###^*P* < 0.001 versus model.

## 3. Results

### 3.1. Wnt/*β*-Catenin, COX-2, and VEGF Were Involved in MK801-Induced Protection of Retinal Neuron Cells from Damage in NMDA-Treated Rats

**Figure 1:**
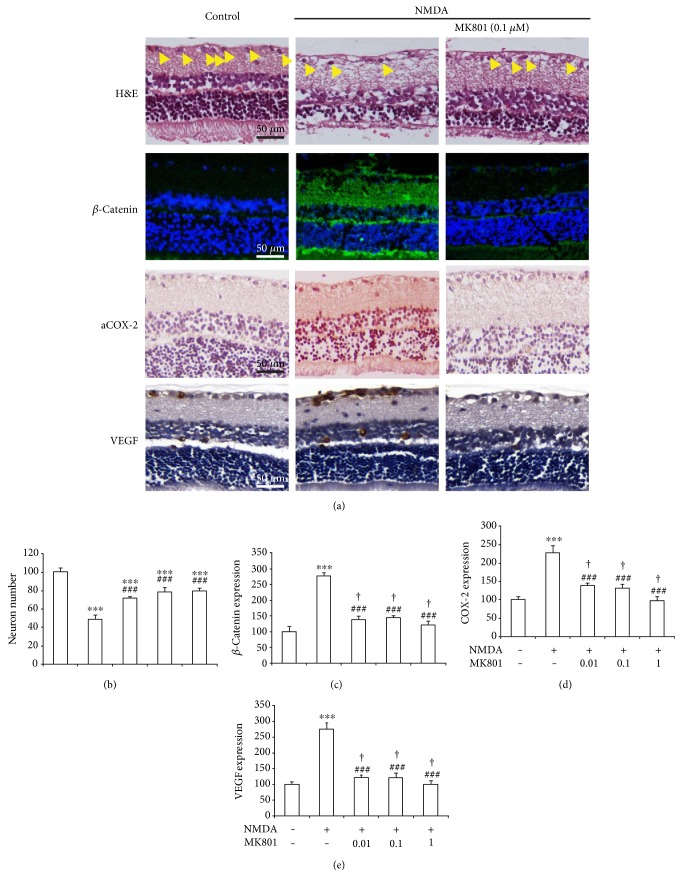
The protection of MK801 on retinal neuron cells in NMDA-treated rats. (a) Representative images (H&E staining, expression of *β*-catenin, COX-2, and VEGF) of the rats. (b) Neuron numbers of RGCs with respect to control (untreated), in NMDA-injected retinas of animals treated with MK801. (c) *β*-Catenin expression of retinas with respect to control in NMDA-injected retinas of animals treated with MK801. (d) COX-2 expression of retinas with respect to control in NMDA-injected retinas of animals treated with MK801. (e) VEGF expression of retinas with respect to control in NMDA-injected retinas of animals treated with MK801.

The neurons in the retinas of normal untreated rats were compact and clear. However, there was an obvious loss of neurons in the retinal ganglion cell layer (GCL) of rats treated with NMDA. The number of neurons in the retinal GCL of the NMDA-treated rats was decreased significantly (49 ± 4% of control) in comparison to that of normal rats (100 ± 5%; Figures 1(a) and 1(b)), indicating that the retinal neurons were damaged by NMDA treatment. In comparison to that of NMDA-treated rats (49 ± 4% of control), the retinal GCL neuron number in the rats treated with NMDA plus MK801 increased in a dose-dependent manner (72 ± 3%, 78 ± 5%, and 79 ± 4% of control, Figure 1(b)), demonstrating that inhibiting the NMDA receptor has a protective effect on the neurons.

Then, we investigated whether the Wnt/*β*-catenin signaling pathway plays a role in the rats' retinal damage induced by NMDA. As shown in Figures 1(a) and 1(c), compared with the retinas of normal rats (100 ± 16%), the retinas of the rats treated with NMDA displayed more abundant *β*-catenin expression in the GCL (278 ± 9% of the control). These data suggest that *β*-catenin accumulation might participate in the NMDA-induced loss of retinal neurons. Then, the effect of MK801 on the expression of *β*-catenin in the rats' retinas was investigated. After the treatment with different doses of MK801 (0.01, 0.1, and 1 *μ*M) in the presence of NMDA, the expression of *β*-catenin in the GCL was decreased significantly (138 ± 12%, 146 ± 4%, and 122 ± 11%, resp.).

As we have indicated that our previous works demonstrated that COX-2 could be an important downstream of *β*-catenin, we detected the expression of COX-2 in retinopathy caused by NMDA. COX-2 expression of the rats' retinas examined by immunohistochemical assays was shown in Figure 1(d). Compared with the normal control rats (100 ± 8%), COX-2 expression of NMDA-treated rats was overexpressed (228 ± 18% of the control). After the treatment with MK801 (0.01, 0.1, and 1 *μ*M) plus NMDA, the overexpressions of COX-2 were reversed (137 ± 7%, 130 ± 12%, and 98 ± 9% of the control, resp.; Figure 1(d)) as compared with those in NMDA-treated rats.

The VEGF expression level was examined by immunohistochemical assays and quantified by a multispectral image analysis. As shown in Figure 1(e), the VEGF expression in the retinal GCL of the NMDA-treated rats was increased in comparison to normal rats. The expressions of VEGF in the NMDA-treated rats' retinas were 276 ± 7% of the control in GCL. In the retinas of the rats treated with MK801 (0.01, 0.1, and 1 *μ*M) plus NMDA, the VEGF expression was lower in the GCL (122 ± 7%, 122 ± 10%, and 101 ± 10% of the control, resp.) than that of the NMDA-treated rats. These results showed that VEGF overexpression could be attenuated by blocking NMDA receptors.

### 3.2. The Wnt/*β*-Catenin Pathway Was Activated in the Retinopathy Caused by NMDA

**Figure 2:**
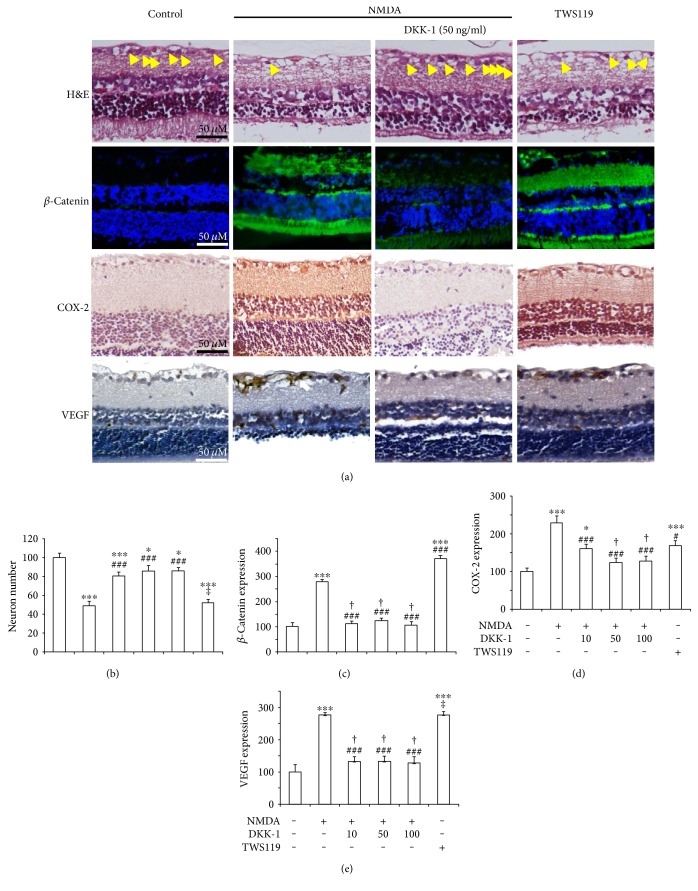
The Wnt/*β*-catenin pathway was activated in the retinopathy caused by NMDA. (a) Representative images (H&E staining, expression of *β*-catenin, COX-2, and VEGF) of the rats. (b) Neuron numbers of RGCs with respect to control (untreated), in NMDA-injected retinas of animals treated with DKK-1. (c) *β*-Catenin expression of retinas with respect to control (untreated), in NMDA-injected retinas of animals treated with DKK-1. (d) COX-2 expression of retinas with respect to control, in NMDA-injected retinas of animals treated with DKK-1. (e) VEGF expression of retinas with respect to control, in NMDA-injected retinas of animals treated with DKK-1.

To further characterized the role of Wnt pathway, or more specifically *β*-catenin, we treated some other rats with intravitreal injections of NMDA plus DKK-1, which is a widely used and effective inhibitor of Wnt/*β*-catenin signal pathway. Immunofluorescent images showed that DKK-1 can decrease the expression of *β*-catenin in retina tissue (Figure 2(a)). The neuron numbers in the retinal GCL of the rats treated with DKK-1 (10, 50, and 100 ng/ml) plus NMDA elevated significantly (81 ± 4%, 86 ± 6%, and 86 ± 4% of control, resp.) compared with those in the NMDA group (49 ± 4% of control; Figure 2(b)), indicating that the inhibition of Wnt/*β*-catenin could block the NMDA-induced loss of neurons. To further investigate the role of *β*-catenin signal in neuronal loss, we utilized TWS119 (a GSK-3*β* inhibitor which inhibited the degradation of *β*-catenin) to induce abnormal *β*-catenin accumulation which mimicked the activation of the canonical Wnt/*β*-catenin signaling pathway [18]. The neuron number in the retinal GCL of TWS119-treated rats was counted, and it was obviously decreased (52 ± 3% of the control), thus indicating that the overexpression of *β*-catenin induced by TWS119 leads to neuronal loss.

The immunofluorescence analyses showed that *β*-catenin was overexpressed in the retinal GCL of NMDA-treated rats (278 ± 9% of the control) and TWS119-treated rats (372 ± 13% of the control). Meanwhile, DKK-1 (10, 50, and 100 ng/ml) plus NMDA attenuated the overexpression of *β*-catenin (122 ± 11%, 124 ± 10%, and 106 ± 13% of the control, resp.; Figure 2(c)). As shown in Figures 2(d) and 2(e), COX-2 was decreased (160 ± 12%, 124 ± 12%, and 128 ± 12% of control, resp.) compared with NMDA-induced rats (228 ± 18% of control), and VEGF was decreased (133 ± 14%, 133 ± 16%, and 127 ± 20% of control, resp.) compared with NMDA-induced rats (228 ± 18% of control; Figure 2(e)), accompanying with low expression of *β*-catenin in the presence of DKK-1 (10, 50, and 100 ng/ml). Meanwhile, with high expression of *β*-catenin (372 ± 13% of control) caused by TWS119, COX-2 was increased (169 ± 14% of control), and VEGF was increased (277 ± 9% of control) as well.

### 3.3. COX-2 Was Involved in the Loss of Neuron Cells Caused by NMDA-Induced Activation of *β*-Catenin

**Figure 3:**
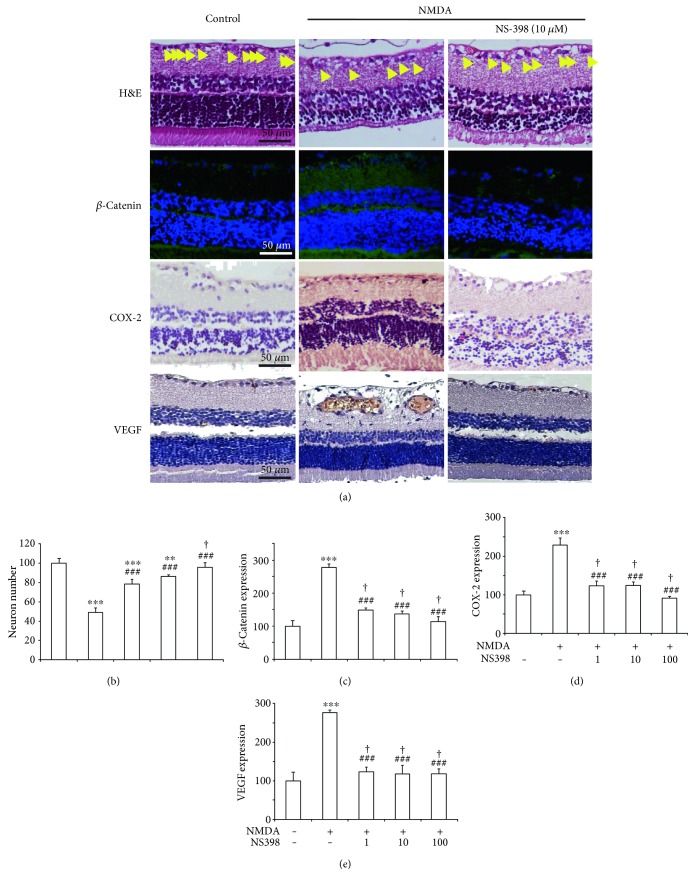
NS-398 could attenuate the NMDA-induced activation of *β*-catenin causing neuron cell loss. (a) Representative images (H&E staining, expression of *β*-catenin, COX-2, and VEGF) of the rats. (b) Neuron number of RGCs with respect to control (untreated), in NMDA-injected retinas of animals treated with NS-398. (c) *β*-Catenin expression of retinas with respect to control in NMDA-injected retinas of animals treated with NS-398. (d) COX-2 expression of retinas with respect to control in NMDA-injected retinas of animals treated with NS-398. (e) VEGF expression of retinas with respect to control in NMDA-injected retinas of animals treated with NS-398.

To further assess the relationship between *β*-catenin and COX-2, we treated some rats with an intravitreal injection of NMDA plus NS-398, an inhibitor of COX-2. The representative image of the rats treated with 10 *μ*Μ NS-398 plus NMDA was shown in Figure 3(a). The neuron numbers in the retinal GCL of the rats treated with NMDA plus NS-398 (10, 50, and 100 ng/ml) were elevated (78 ± 5%, 86 ± 2%, and 96 ± 4% of control, resp.) compared with those in the NMDA-treated group (49 ± 4% of control). This demonstrated that the inhibition of COX-2 could block the NMDA-induced loss of neurons, which may also contribute to retinal protection (Figure 3(b)).

**Figure 4:**
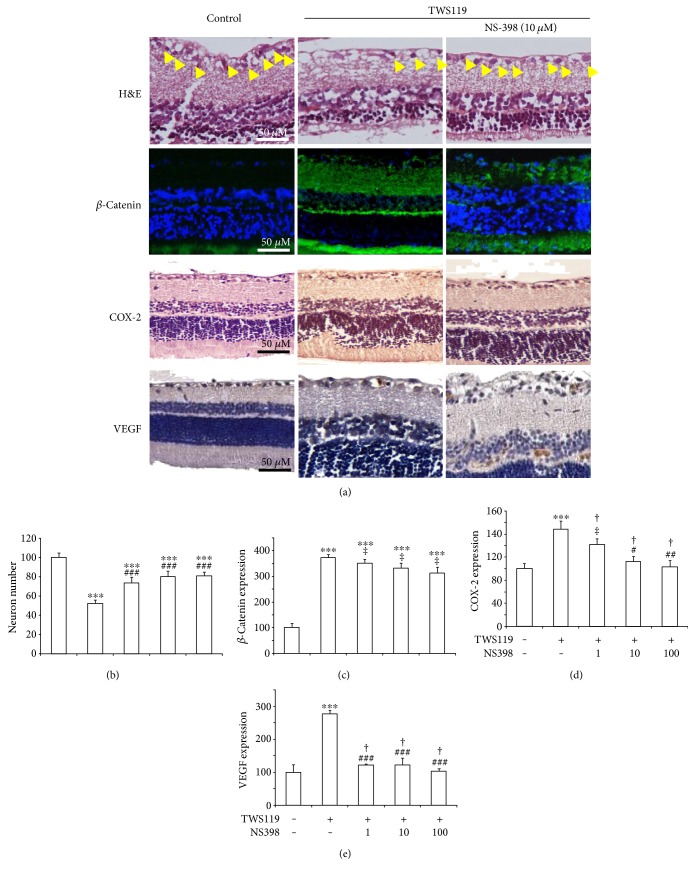
NS-398 could attenuate the TWS119-induced activation of *β*-catenin causing neuron cell loss. (a) Representative images (H&E staining, expression of *β*-catenin, COX-2, and VEGF) of the rats. (b) Neuron number of RGCs with respect to control (untreated), in TWS119-injected retinas of animals treated with NS-398. (c) *β*-Catenin expression of retinas with respect to control in TWS119-injected retinas of animals treated with NS-398. (d) COX-2 expression of retinas with respect to control in TWS119-injected retinas of animals treated with NS-398. (e) VEGF expression of retinas with respect to control in TWS119-injected retinas of animals treated with NS-398.

In addition, the neuron numbers in the retinal GCL of the rats treated with NS-398 (10, 50, and 100 ng/ml) plus TWS119 elevated (73 ± 6%, 80 ± 6%, and 80 ± 4% of control, resp.) compared with those in the TWS119 group (52 ± 3% of control). This indicated that the inhibition of COX-2 could block the TWS119-induced loss of neurons, which may contribute to retinal protection (Figure 4(b)).

Then, we detected the expression of *β*-catenin with NS398 (1, 10, and 100 *μ*M) plus NMDA, and we found that the expression of COX-2 in NMDA plus NS-398 (124 ± 11%, 124 ± 8%, and 91 ± 4% of control, resp.) was lower compared with the NMDA group (228 ± 18% of control; Figure 3(d)). In the same trend, *β*-catenin was decreased (148 ± 7%, 137 ± 8%, and 114 ± 13% of control, resp.; Figure 3(c)), compared with the NMDA group (278 ± 9% of control); VEGF was decreased (122 ± 18%, 117 ± 22%, and 118 ± 12% of control, resp.; Figure 3(e)), compared with the NMDA group (276 ± 7% of control).

While the expression of *β*-catenin (350 ± 16%, 350 ± 16%, and 332 ± 18% of control, resp.) was unchanged in TWS119 plus NS-398 compared with TWS119 group (372 ± 13%; Figure 4(c)), COX-2 was attenuated (141 ± 10%, 113 ± 8%, and 102 ± 12% of control, resp.) in TWS119 plus NS-398 (1, 10, and 100 *μ*M; Figure 4(d)), and VEGF was decreased (121 ± 3%, 122 ± 20%, and 102 ± 7% of control, resp.; Figure 4(e)).

### 3.4. VEGF Was the Downstream of Wnt/*β*-Catenin Signaling and COX-2 in Retinopathy Caused by NMDA

**Figure 5:**
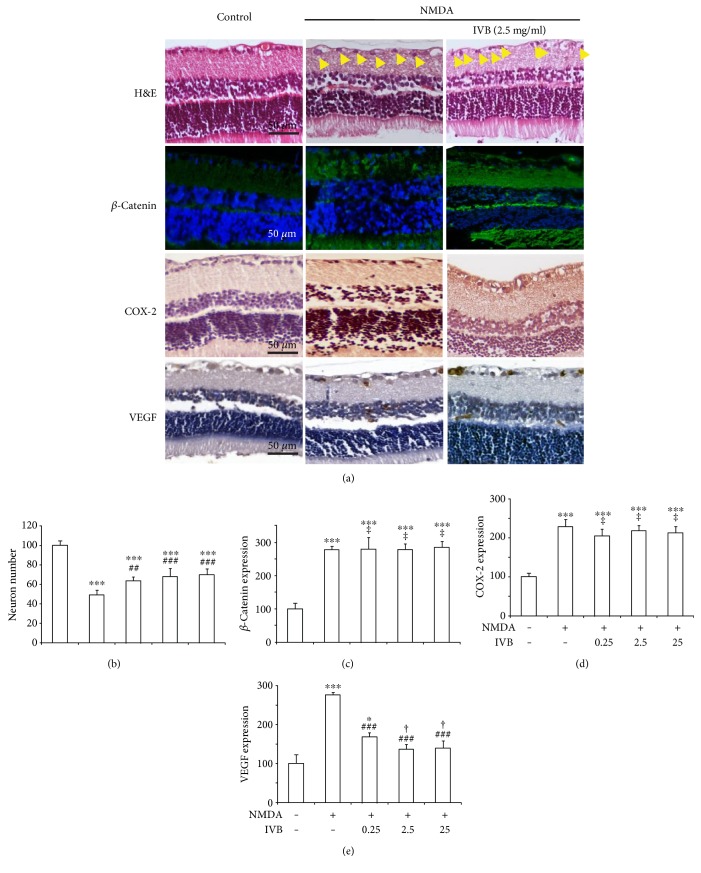
IVB could ameliorate retinopathy caused by NMDA through reducing VEGF expression (a) Representative images (H&E staining, expression of *β*-catenin, COX-2, and VEGF) of the rats. (b) Neuron number of RGCs with respect to control (untreated), in NMDA-injected retinas of animals treated with IVB. (c) *β*-Catenin expression of retinas with respect to control in NMDA-injected retinas of animals treated with IVB. (d) COX-2 expression of retinas with respect to control in NMDA-injected retinas of animals treated with IVB. (e) VEGF expression of retinas with respect to control in NMDA-injected retinas of animals treated with NMDA.

Bevacizumab (IVB), an anti-VEGF antibody that could inhibit VEGF, was used to further characterize the retinopathy caused by NMDA. As shown in Figures 5(a) and 5(b), the neuron numbers in the retinal GCL of the rats treated with IVB (0.25, 2.5, and 25 mg/ml) plus NMDA elevated (64 ± 3%, 68 ± 8%, and 70 ± 5% of control, resp.) compared with those in the NMDA group (49 ± 4% of control). This indicated that the inhibition of VEGF could at least partially prevent the NMDA-induced loss of neurons, which may contribute to retinal protection. In accordance, Figure 5(e) presented that the IVB (0.25, 2.5, and 25 mg/ml) treatment decreased the expression of VEGF in the retinas of rats treated with NMDA (168 ± 11%, 137 ± 11%, and 140 ± 18% of control, resp.) compared with NMDA-treated rats (276 ± 7%). In the contrary, Figures 5(c) and 5(d) demonstrated that IVB (0.25, 2.5, and 25 mg/ml) treatment could affect the expression of neither *β*-catenin (280 ± 9%, 278 ± 17%, and 283 ± 19% of control, resp.) nor COX-2 (205 ± 17%, 219 ± 13%, and 213 ± 16% of control, resp.) in the retinas of rats treated with NMDA (278 ± 9% and 220 ± 18%). These findings demonstrate that the inhibition of VEGF did not modulate the *β*-catenin and COX-2 overexpression induced by NMDA, although the inhibition of VEGF helped attenuate the loss of neurons.

**Figure 6:**
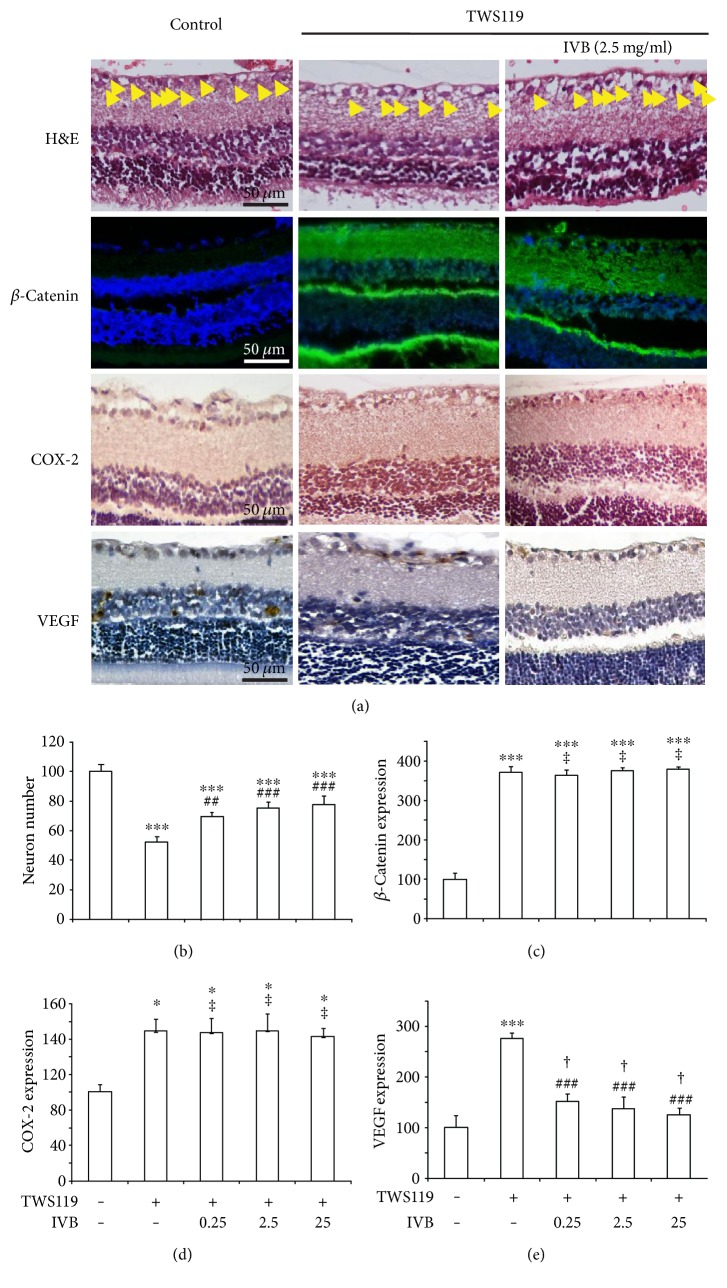
IVB could ameliorate retinopathy caused by TWS119 through reducing VEGF expression (a) Representative images (H&E staining, expression of *β*-catenin, COX-2, and VEGF) of the rats. (b) Neuron number of RGCs with respect to control (untreated), in TWS119-injected retinas of animals treated with IVB. (c) *β*-Catenin expression of retinas with respect to control in TWS119-injected retinas of animals treated with IVB. (d) COX-2 expression of retinas with respect to control in TWS119-injected retinas of animals treated with IVB. (e) VEGF expression of retinas with respect to control in TWS119-injected retinas of animals treated with TWS119.

To evaluate the relationship between activation of Wnt pathway and VEGF, the VEGF expression level of TWS119-treated retinas in rats was examined by immunohistochemical assays. We could see from Figure 6(b) that an intravitreal injection of TWS119 plus IVB (0.25, 2.5, and 25 mg/ml) increased the number of retinal GCL neurons (70 ± 3%, 75 ± 4%, and 78 ± 6% of control, resp.) in the rats treated with TWS119 (52 ± 3% of the control). Statistical analyses also showed that the IVB (0.25, 2.5, and 25 mg/ml) treatment decreased the expression of VEGF in the retinas of rats treated with TWS119 (151 ± 14%, 138 ± 22%, and 125 ± 12% of control, resp.) compared with TWS119-treated rats (277 ± 23%), as shown in Figure 6(e). Moreover, IVB failed to inhibit *β*-catenin expression levels in the retinal GCL of TWS119-treated rats (364 ± 12%, 375 ± 7%, and 379 ± 6% of control, resp.). The expression of COX-2 in the retinas of TWS119-treated rats was also unchanged after treatment with IVB (Figure 6(d)). Statistical analyses show that the IVB (0.25, 2.5, and 25 mg/ml) treatment decreased the expression of COX-2 in the retinas of rats treated with TWS119 (167 ± 16%, 169 ± 19%, and 163 ± 9% of control, resp.) compared with TWS119-treated rats (169 ± 14% of control).

### 3.5. Discussion

**Figure 7:**
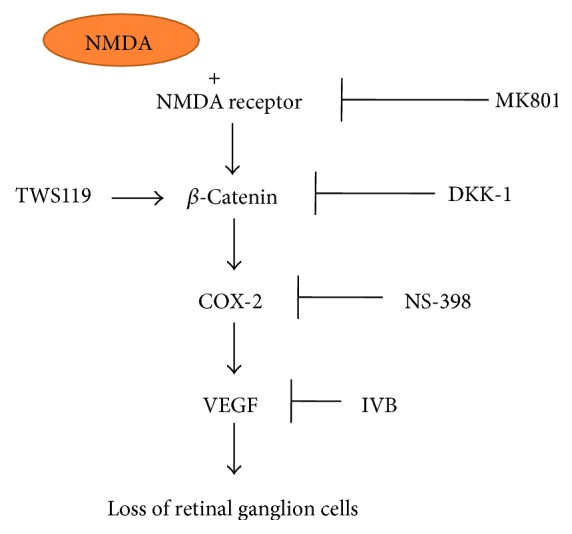
Schematic graph illustrating the involvement of Wnt/*β*-catenin, COX-2, and VEGF in retinopathy induced by NMDA.

The present study demonstrates that the *β*-catenin signaling pathway is activated by NMDA in the retinas of rats and for the first time that the activation of the *β*-catenin signaling pathway is involved in the overexpression of COX-2 and VEGF, which leads to retinal ganglion cell loss in the retinal GCL of rats treated with NMDA. Furthermore, we have shown that DKK-1, NS-398, and IVB can ameliorate retinal ganglion cell loss, suggesting that the *β*-catenin/COX-2/VEGF axis plays a causative role in retinopathy treated with NMDA. Therefore, these observations have established a new mechanism for the NMDA-induced retinal ganglion cell loss (Figure 7).

Excessive activation of NMDA receptors *in vitro* and *in vivo* might directly cause the degeneration of retinal neurons which due to the large amount of NMDA receptors present on the membranes of retinal GCL neurons [19–21]. Our present study revealed that there was extensive neuronal loss after intravitreal injection of NMDA. The activation of the NMDA receptor was thought to be insufficient to explain the neuronal loss [22, 23]. In the present study, we found that cotreatment with MK801 (100 nM or 1 *μ*M) could decrease the NMDA-induced neuron loss. The activation of the Wnt/*β*-catenin signaling pathway was previously found in injured retinal tissues *in vitro* [24]. In addition, an inhibitor (Dickkopf-1) of the Wnt/*β*-catenin signaling pathway could alleviate diabetic retinopathies [18, 25]. To evaluate the activation of the Wnt/*β*-catenin pathway in NMDA-induced retinal damage in rats, we examined retinal *β*-catenin levels in the NMDA-damaged retinas of rats. The immunofluorescence analyses demonstrated abnormal accumulation of *β*-catenin in the retinal GCL of the NMDA-treated rats in comparison to that of normal rats. COX-2 is one of the target genes and a modulating factor of the Wnt/*β*-catenin pathways, we checked the COX-2 expression and found that COX-2 increased after NMDA treatment. In addition, VEGF was increased after NMDA treatment. Notably, the inhibition of the NMDA receptor by MK801 could decrease the expression levels of *β*-catenin, COX-2, and VEGF (Figure 1). These findings have shown that the inhibitors of NMDA can protect retinal ganglion cells through decreasing the expression of *β*-catenin, COX-2, and VEGF.

Since Wnt pathway is known to be activated under retinopathy conditions [24], we further demonstrated the causative role of activated Wnt signaling in retinal caused by NMDA. We blocked the Wnt pathway by using DKK-1, a specific peptide inhibitor of the Wnt pathway. Intravitreal injection of NMDA plus DKK-1 is sufficient to mitigate retinal inflammation as it blocks the overexpression of *β*-catenin, which is the upstream of COX-2. Similarly, DKK-1 also reduced the ischemia-induced retinal neovascularization, the VEGF expression. These results indicate that blockade of the Wnt pathway is sufficient to ameliorate the damage caused by NMDA. Further, we mimicked the activation of the *β*-catenin pathway using TWS119. By inhibiting GSK-3*β* activation, TWS119 leads to the accumulation of *β*-catenin in the cytoplasm, which subsequently translocates to the nucleus [15]. Our immunofluorescence data demonstrated that either VEGF or COX-2 was overexpressed in the GCL of the TWS119-treated rats. Moreover, we found that the TWS119-treated rats suffered more serious neuronal loss than the control rats did (Figure 2). These results indicate that stimulation of the *β*-catenin signaling pathway could induce neuronal loss. Further, activation of the *β*-catenin signaling pathway, without NMDA treatment, was sufficient to induce COX-2, VEGF expression, and neuronal loss.

In the present study, the expression levels of both COX-2 and VEGF increased after the administration of either NMDA or TWS119. Therefore, COX-2 and VEGF might be regulated by the activation of the *β*-catenin pathway in damaged retinas. We used specific inhibitors to examine the interaction between these various molecules.

NS-398, an inhibitor of COX-2, could attenuate the loss of retinal ganglion cells. Interestingly, inhibiting COX-2 by NS398 could decrease the NMDA-induced *β*-catenin overexpression that shows that COX-2 maybe upstream of *β*-catenin, which was confirmed by Li T that COX-2 can promote hepatocarcinogenesis through activation of *β*-catenin signaling pathway [17]. Similarly, inhibiting COX-2 by NS398 could attenuate the NMDA-induced VEGF overexpression (Figure 3). However, in TWS119-treated rats, NS398 failed to inhibit the activation of *β*-catenin. This is in accordance with our previous work [10] that there could be a feedback loop between *β*-catenin and COX-2.

These findings have shown that COX-2 is upstream of *β*-catenin and maybe upstream of VEGF. We are continuing our investigations to further elucidate how COX-2 signaling regulates *β*-catenin and VEGF. We found that NS398 was administered with TWS119 which could protect retinal ganglion cell from losing. However, NS398 could not reverse the TWS119-induced *β*-catenin overexpression but could attenuate VEGF overexpression caused by TWS119 (Figure 4()). This further demonstrated that VEGF maybe downstream of COX-2.

IVB is an anti-VEGF agent used to treat diabetic macular edema. In this study, we found that treatment with IVB could not reverse the NMDA- or TWS119-induced COX-2 and *β*-catenin overexpression (Figures 5() and 6()). But IVB can protect retinal ganglion cells treated with NMDA or TWS119. These results demonstrate inhibiting VEGF did not interfere with the *β*-catenin and COX-2 expression suggesting that VEGF is downstream of COX-2, which was proved in gastric cancer [16].

In summary, the present study provides the first evidence showing that activation of the *β*-catenin/COX-2/VEGF signaling pathway is an important pathogenic mechanism underlying the NMDA-induced retinal damage in animal models. Thus, the *β*-catenin/COX-2/VEGF pathway might represent a new target for pharmaceutical intervention for retinal diseases.
